# Tumor Protein (TP)-p53 Members as Regulators of Autophagy in Tumor Cells upon Marine Drug Exposure

**DOI:** 10.3390/md14080154

**Published:** 2016-08-16

**Authors:** Edward A. Ratovitski

**Affiliations:** Head and Neck Cancer Research Division, Johns Hopkins University School of Medicine, Baltimore, MD 21231, USA; eratovi1@jhmi.edu; Tel.: +1-410-491-9802; Fax: +1-410-614-1411

**Keywords:** marine drugs, cancer, autophagy, p53 family members, transcription

## Abstract

Targeting autophagic pathways might play a critical role in designing novel chemotherapeutic approaches in the treatment of human cancers, and the prevention of tumor-derived chemoresistance. Marine compounds were found to decrease tumor cell growth in vitro and in vivo. Some of them were shown to induce autophagic flux in tumor cells. In this study, we observed that the selected marine life-derived compounds (Chromomycin A2, Psammaplin A, and Ilimaquinone) induce expression of several autophagic signaling intermediates in human squamous cell carcinoma, glioblastoma, and colorectal carcinoma cells in vitro through a transcriptional regulation by tumor protein (TP)-p53 family members. These conclusions were supported by specific qPCR expression analysis, luciferase reporter promoter assay, and chromatin immunoprecipitation of promoter sequences bound to the TP53 family proteins, and silencing of the TP53 members in tumor cells.

## 1. Introduction

Autophagy is a cellular mechanism underlying elimination of denatured cytoplasmic proteins, damaged protein aggregates, and organelles through the formation of autophagosome vesicles with subsequent transfer of their cargo into the lysosomes for degradation [1]. Autophagy is increased when cells are under stress conditions, such as nutritional deficiency, DNA damage, hypoxia, and other stressors [1,2,3]. Autophagy can potentially modulate the pro-survival and pro-death mechanisms in tumor initiation and progression, as reviewed in [1,2,3]. Defective autophagy might contribute to tumorigenesis via accumulation of damaged mitochondria and protein aggregates, leading to a production of reactive oxygen species and causing DNA damage, and subsequently genomic instability [1,2,3]. Manipulating autophagy for induction of cell death, inhibition of protective autophagy, and its crosstalk with tissue-specific apoptosis might yield promising anticancer chemotherapeutic venues [4,5]. The tumor suppressive function of autophagy has been often reported, as reviewed in [3,6,7].

Tumor protein (TP)-p53, TP63, and TP73 transcription factors represent proteins with high structural and functional similarities implicated in the regulation of numerous gene targets upon multiple stress conditions [8]. They regulate various signaling pathways leading to cell death and cell survival, cell cycle arrest and cell proliferation, cellular metabolic cascades, and cell differentiation [8]. TP53 family proteins often overlap in their ability to activate/inhibit transcription of specific target genes with various functions [8]. Accumulating evidence shows that all TP53 family proteins (TP53, TP63, and TP73) are implicated in the regulation of autophagic responses by tumor cells to distinct extracellular challenges, often leading to cell survival but sometimes just delaying inevitable tumor cell death upon stress, such as chemotherapeutic treatment [9,10]. Activation of autophagy by TP53 proteins suggests that autophagy is part of the protective function of TP53 and other members of this family [9,10,11,12,13]. Uncovering the underlying mechanisms of the autophagy-TP53 reciprocal functional interaction has important implications for human disease and treatment [11,12,13]. Mounting evidence clearly suggests that the TP53 family members regulate a network of genes implicated in cell death, and may enhance or fine-tune the autophagic response upon stress stimuli [14,15,16]. TP53 family nuclear transcription factors transactivate pro-apoptotic, cell cycle-arresting, and pro-autophagic genes [13,17]. Genotoxic stress can induce autophagy in a TP53-dependent fashion through a transcriptional activation of autophagy-inducing genes [13]. The critical regulators of autophagy, DRAM1 (damage-regulated autophagy modulator), BECN1, SESTRIN2, and ATG1/ULK1 (autophagy-initiation kinase) are transcriptionally activated by TP53 and subsequently induce autophagic pathways [14,15,16,18,19,20,21,22]. TP53-induced autophagy may lead to cell death, which can be blocked by *DRAM1* siRNA [18,20,21].

Recently, the AEN/ISG20L1 protein was identified as a TP53-dependent, genotoxic stress-induced modulator of autophagy [23]. Transcription of the *AEN* gene can be regulated by all three TP53 family members (TP53, TP63, and TP73) and *AEN* knockdown decreases levels of autophagic vacuoles and LC3B-II protein after genotoxic stress, strengthening the connection between TP53 signaling and autophagy [23]. Several pro-apoptotic genes, including TP53-upregulated modulator of apoptosis protein (*PUMA*) and BCL-2-associated X protein (*BAX*) are also positive regulators of autophagy (e.g., mitochondrial autophagy), as reviewed elsewhere [24].

Endogenous TP73 protein was found to bind the regulatory regions of specific autophagic genes, such as *ATG5*, *ATG7*, and *UVRAG*, as indicated in [17]. Although TP73 was found to induce expression of *ATG5* and *ATG7* genes, TP73 knockdown increased the *UVRAG* expression levels [17,25]. The TP53 homolog TP63 is a novel transcription factor implicated in the regulation of genes involved in DNA damage response and chemotherapeutic stress in tumor cells [26]. The TP63 gene encodes two types of protein isotypes, with the long transactivation (TA)-domain and with the short TA-domain (known as ΔN-), as reviewed in [26]. The ΔNp63α is the most predominantly expressed isotype in head and neck squamous cell carcinoma (SCC) cells [27]. ΔNp63α was shown to activate ATM transcription, thereby contributing to the ATM-TSC2-mTOR complex 1-dependent autophagic pathway [28,29]. ΔNp63α was shown to transcriptionally regulate the expression of the members of the autophagic pathway, such as *ATG1/ULK1, ATG3, ATG4A, ATG5, ATG6/BECN1, ATG7, ATG9A,* and *ATG10* genes, as described elsewhere [30].

Targeting autophagic pathways might play a critical role in designing novel chemotherapeutic approaches in the treatment of human cancers, and the prevention of tumor-derived chemoresistance, as reviewed in [4,5,16]. Natural products from plants, fungi, and marine organisms could play a promising role in the development of novel anticancer chemotherapeutics [2,31,32,33,34,35,36]. Accumulating evidence shows that many anticancer compounds could be isolated from marine organisms, including bacteria, actinomycetes, sponges, etc. [37,38,39,40,41,42,43,44]. Some of them show dramatic effects on various human cancer cells in vitro, as well as in vivo, and a few displayed success in preclinical studies [39]. Anticancer marine compounds often induce cell cycle arrest, apoptosis, and autophagy, thereby hindering tumor cell survival in vitro and in vivo [40,41,42,43,44]. The molecular mechanisms underlying the cytotoxic functions of marine compounds toward a variety of tumor cells is largely unclear, therefore molecular studies could enhance our understanding of the specific targets for various marine compounds in human tumor cells. The role for tumor protein (TP)-p53 family members (TP53, TP63, and TP73), as master regulators of genome integrity through transcription and other molecular processes, could not be more emphasized. These proteins are involved in a myriad of cellular processes (cell cycle arrest, apoptosis, autophagy, necroptosis, etc.) affecting tumor cell survival, and could clearly be critical molecular targets for anticancer therapies [6,13,14,16]. Upon treatment with various anticancer agents, tumor cells often undergo DNA damage leading to activation of TP53 family members through a specific mechanism of protein phosphorylation [13,26,28]. Thus, we chose to investigate the molecular response of these proteins to the marine drug treatment in cancer cells.

Many marine compounds have been successfully used in the inhibition of tumor cell growth in vitro and in vivo [37,38,39,40]. Among them, special attention was given to compounds that are able to induce autophagic flux in tumor cells [41,42,43,44,45]. This work is an attempt to connect selected marine compounds (Chromomycin A2, Psammaplin A, and Ilimaquinone), with autophagic signaling intermediates and TP53 family transcriptional regulators in various human tumor cells (squamous cell carcinoma, glioblastoma, and colorectal carcinoma), to understand and define molecular mechanisms underlying their cooperation in modulation of tumor cell survival upon treatment.

## 2. Results

### 2.1. Marine Compounds Decrease Tumor Cell Viability in a Dose- and Time Dependent Manner

**Figure 1:**
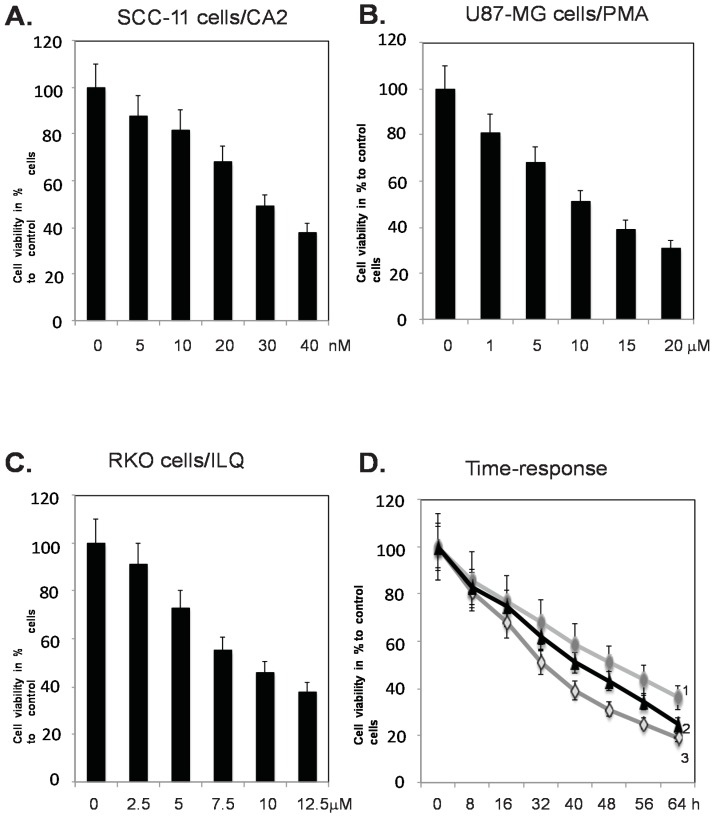
Selected marine drugs decrease tumor cell viability in dose- and time-dependent manner. Tumor cells (SCC-11, **panel A**, U87-MG, **panel B**, and RKO, **panel C**) were treated with control media and chromomycin A2 (CA2), psammaplin A (PMA), and ilimaquinome (ILQ), respectively in a dose-dependent manner for 24 h, as indicated. Tumor cell viability is shown as a percentage of viable cells after drug treatment compared to control media treatment. In panel **D**, tumor cells (SCC-11, designated as 1, U87-MG, designated as 2, and RKO, designated as 3) are treated with control media and CA2 (30 nM), PMA (7.5 µM), and ILQ (10 µM) for the indicated time periods.

For the current study, we selected three cell lines derived from human cancers; squamous cell carcinoma (SCC-11), glioblastoma (U87-MG), and colon colorectal cancer (RKO). These tumor cell lines are known to predominantly express TP63 (ΔNp63α isoform for SCC-11), TP73 (U87-MG), and TP53 (RKO), and were available in our laboratory [27,46,47]. The marine compounds selected for these studies were Chromomycin A2 (CA2), Psammaplin A (PMA), and Ilimaquinone (ILQ). All these compounds are commercially available and have been previously reported to induce autophagy in tumor cells [40,42,43], as well as affect expression of TP53 and its posttranslational modifications [40,43], therefore strengthening thepotential role of other TP53 family proteins, which are likely contributing to drug-induced autophagy. We first tested the effect of selected marine anticancer compounds on the viability of tumor cells using the MTT assay, as described in the Materials and Methods section. Our initial experiments showed that the tested marine anticancer compounds (CA2, PMA, and ILQ) decreased the cell viability of selected tumor cells in a dose-dependent manner (Figure 1A–C), as well as in a time-dependent manner (Figure 1D). We found that ~50% of SCC-11 cells had died upon treatment with ~30 nM CA2, while ~50% of U87-MG cells had undergone cell death upon treatment with 7.5 µM PMA, and ~50% of RKO cells had not survived upon exposure to 10 µM ILQ (Figure 1A–C). We further found that the selected concentrations of the tested marine compounds (CA2, PMA, and ILQ) dramatically decreased the viability of tumor cells (SCC-11, U87-MG, and RKO) between 12 and 24 h (Figure 1D).

These data were used to build a foundation for the subsequent molecular analysis of the expression of TP53 family proteins and their autophagic target genes. Thus, we chose to treat selected tumor cells with 30 nM CA2 for SCC-11 cells, 7.5 µM PMA for U87-MG cells, and 10 µM ILQ for RKO cells. Since TP53 family members could transcriptionally regulate many autophagic genes and often overlap in their function [10,11,12,13,14,15,16], we selected two distinct representative autophagy genes for the specific expression of each TP53 member protein in tested tumor cell lines, such as TP63—for SCC-11 cells, TP73—for U87-MG cells, and TP53—for RKO cells [27,45,46].

### 2.2. Marine Compounds Induce Expression and Phosphorylation of TP53 Family Members in Human Tumor Cells

**Figure 2:**
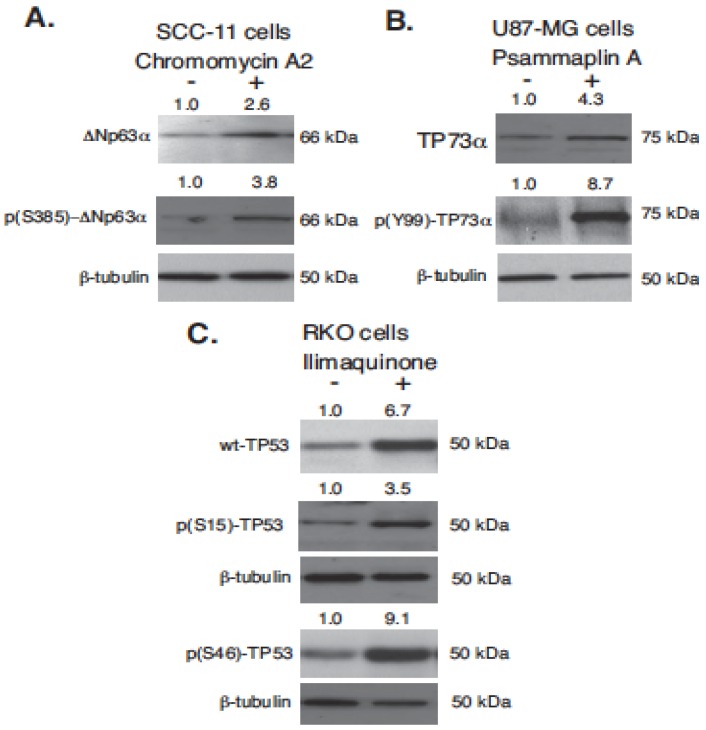
Selected marine drug activated p53 family members in human tumor cells. Tumor cells (SCC-11, M87-MG, and RKO) were treated with control media (−) and chromomycin A2 (+, 30 nM, **panel A**), psammaplin A (+, 7.5 µM, **panel B**) and ilimaquinone (+, 10 µM, **panel C**) for 16 h. Total protein lysates were analyzed by immunoblotting with indicated antibodies against ΔNp63α TAp73α and wild type (wt)-TP53, as well as against p(S385)-ΔNp63α, p(Y99)-TP73, p(S15)-TP53, and p(S46)-TP53. Level of β-tubulin served as a loading control.

The next step of our study was to evaluate the expression of TP53 family proteins and their phosphorylation in SCC-11, U87-MG, and RKO tumor cells upon exposure to 30 nM CA2, 7.5 µM PMA, and 10 µM ILQ, respectively. We found that the selected marine anticancer compounds led to a marked increase (2.6–6.7 × fold) in the protein levels for TP63 (ΔNp63α), TP73 (TP73α), and TP53 (wild type p53), as shown in Figure 2. We further found that the tested marine compounds dramatically induced the phosphorylation of TP53 family members at specific positions (3.8–9.1 × fold, Figure 2). While phosphorylation of TP53 family members is often critical for the activation of TP53 proteins as transcription factors, the specific phosphorylation events (S385 for ΔNp63α Y99 for TP73α, and S15 and S46 for TP53) have also been found to serve as biomarkers underlying the role of TP53 family members in reducing tumor cell survival and inducing cell death via multiple mechanisms [29,30,45]. Thus, we found that these phosphorylation mechanisms were also induced in tested tumor cells (SCC-11, U87-MG, and RKO) upon exposure to marine anticancer compounds (CA2, PMA, and ILQ).

### 2.3. Marine Compounds Induce Autophagic Flux in Human Tumor Cells

Emerging evidence shows that the autophagy pathway is one of the molecular mechanisms leading to the modulation of tumor cell survival [1,3]. Although autophagy can serve as a pro-survival phenomenon, it often delays the death of tumor cells via apoptosis, essentially contributing to the demise of tumor cells upon treatment with anticancer compounds of various origins [3,6,7,8]. Previous reports and reviews showed that marine anticancer drugs were widely known to induce autophagy [40,41,42,43,44]. TP53 family proteins were found to play a critical role in autophagy signaling, as widely reported by several groups [10,11,12,14,15,16,17,18,19,20,21,22,23,24,25,29,30]. TP53 proteins were shown to induce multiple molecular pathways, including transcriptional activation of genes targeting autophagic machinery [14,17,18,19,20,21,22,23,24,25,29,30].

**Figure 3:**
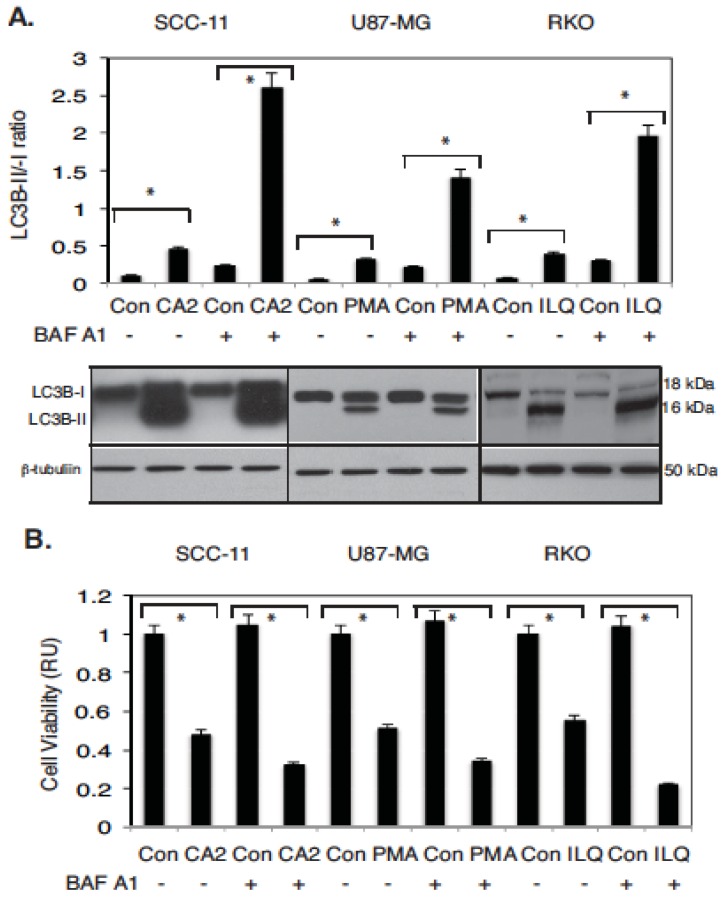
Selected marine drugs induced autophagic flux and inhibit cell viability in tumor cells. Tested tumor cells were treated with control media (Con), or chromomycin A2 (CA2, 30 nM), psammaplin A (PMA, 7.5 µM) and ilimaquinone (ILQ, 10 µM) for 48 h. Additionally, the 100 nM of bafilomycin A1 (BAF A1, #B1793, Sigma) was added to cells for 12 h, as indicated by (+). (**A**) Protein levels for LC3B-I, -II, normalized by the β-tubulin level (loading control) were examined using immunoblotting (representative immunoblots shown). Immunoblots were scanned using PhosphorImager (Molecular Dynamics) and quantified by ImageQuant software version 3.3 (Molecular Dynamics). Values of LC3B-II were expressed as a portion of LC3B-I values defined as 1. The LC3B-II/LC3B-I ratios were plotted as bars using the Microsoft Excel software with standard deviations (± SD) resulting from three independent experiments and three individual measurements of each experiment (* *p* < 0.05, *t*-test); (**B**) Cell viability assay. 10^4^ cells/well in 96-well plates were incubated in serum-free medium with 5 μg/mL of 3-(4,5-dimethyl thiazol-2-yl)-2,5-diphenyl tetrazolium bromide (American Tissue Culture Collection) in the dark for 4 h at 37 °C. Cells were lysed and incubated for 2 h at 37 °C, and the measurements (A_570nm_ to A_650nm_) were obtained on a Spectra Max-250 plate reader (Molecular Devices), as described in reference 44. Each assay was repeated three times in triplicate. Diagrams indicated the extent of cell viability expressed as a portion of the control represented as 1. Bars are the mean ± SD of triplicate (* *p* < 0.05, *t*-test).

In the current study, we analyzed the effect of selected marine compounds on autophagic flux through their ability to induce the conversion of the autophagic marker LC3B from a cytosolic LC3B-I form to a phosphatidyl-ethanolamine-conjugated LC3B-II form, as reviewed in [29,30]. To further support the role of marine compounds in induction of autophagic flux, tumor cells were grown in the absence or presence of bafilomycin A1 (BAF A1), as indicated in [29,30]. We first found that the treatment with CA2, PMA, and ILQ lead to a marked increase in the LC3B-II/LC3B-II ratio in tested tumor cells (up to 2.8–3.2 × fold). We further found that the additional exposure to BAF A1 of selected tumor cells pre-treated with CA2, PMA, and ILQ significantly increased the LC3B-II protein levels and the LC3B-II/-I ratio (up to 4.3–10.1 × fold), as shown in Figure 3A.

We further showed that the tested marine compounds in contrast to the control media markedly decreased the tumor cell viability, as demonstrated in Figure 3B. We also showed that BAF A1 dramatically decreased the viability of SCC-11, U87-MG, and RKO tumor cells used in these studies, supporting a potential role for autophagic flux in drug-dependent tumor cell death (Figure 3B).

### 2.4. Marine Compounds Activate Transcription of Autophagic Genes through TP53 Family Member’s Transcriptional Function

**Figure 4:**
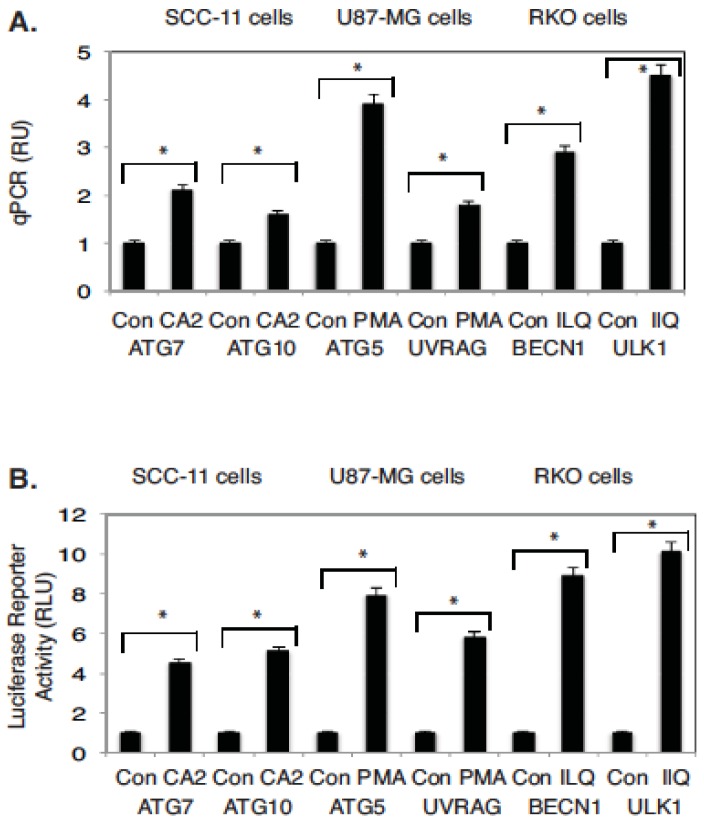
Expression of autophagic genes (*ATG7, ATG10, ATG5, UVRAG, BECN1,* and *ULK1*) in tumor cells treated with marine drugs. (**A**) Expression of indicated autophagic genes (*ATG7, ATG10, ATG5, UVRAG, BECN1,* and *ULK1*) in tumor cells upon exposure to marine drugs. QPCR analysis of gene targets in SCC-11 cells, U87-MG cells, and RKO cells. Three independent qPCR assays were performed in triplicate (± SD are indicated. * *p* < 0.05); (**B**) Luciferase reporter assay of target gene promoters (*ATG7, ATG10, ATG5, UVRAG, BECN1,* and *ULK1*). Tested cells were transfected for 24 h with 100 ng of the LightSwitch_Pro reporter plasmids containing the indicated autophagic promoters or with 100 ng of the control promoter-less reporter plasmid. Cells were exposed to control medium (Con) or medium with chromomycin A2 (CA2, 30 nM), psammaplin A (PMA, 7.5 µM), and ilimaquinone (ILQ, 10 µM) for 12 h. RenSP Renilla luciferase reporter activity assays were conducted using three independent biological experiments in triplicate (± SD are indicated. * *p* < 0.05). Data is presented as relative to the data obtained from the control untreated cells containing the promoter-less reporter plasmid designated as 1.

We further analyzed the effect of selected marine anticancer compounds (CA2, PMA, and ILQ) on the molecular targets, which are transcriptionally regulated by TP53 family members, and are implicated in autophagy signaling [14,17,30]. Using a real-time quantitative PCR, we found that in contrast to control media, CA2 markedly induced the expression levels of *ATG7* and *ATG10* in SCC-11 cells, PMA- of *ATG5* and *UVRAG* in U87-MG cells, and ILQ- of *BECN1* and *ULK1* in RKO cells (Figure 4A).

To examine the functional activity of autophagic gene promoters in tumor cells upon exposure to marine anticancer drugs, we used the LightSwitch Pro Reporter System, which allows monitoring of the Renilla luciferase reporter activity driven by the tested promoters. Thus, selected tumor cells (SCC-11, U87-MG, and RKO) previously transfected with the luciferase reporter plasmids carrying promoter sequences for *ATG7, ATG10, ATG5, UVRAG, BECN1, ULK1,* or control luciferase plasmid were treated with CA2, PMA, or ILQ, as indicated in Figure 4B. The luciferase reporter assay revealed that in contrast to the control, CA2 dramatically induced the promoter activities for *ATG7* and *ATG10*, PMA induced the *ATG5* and *UVRAG* promoter activities, while ILQ induced the activities for *BECN1* and *ULK1* promoters (Figure 4B).

**Figure 5:**
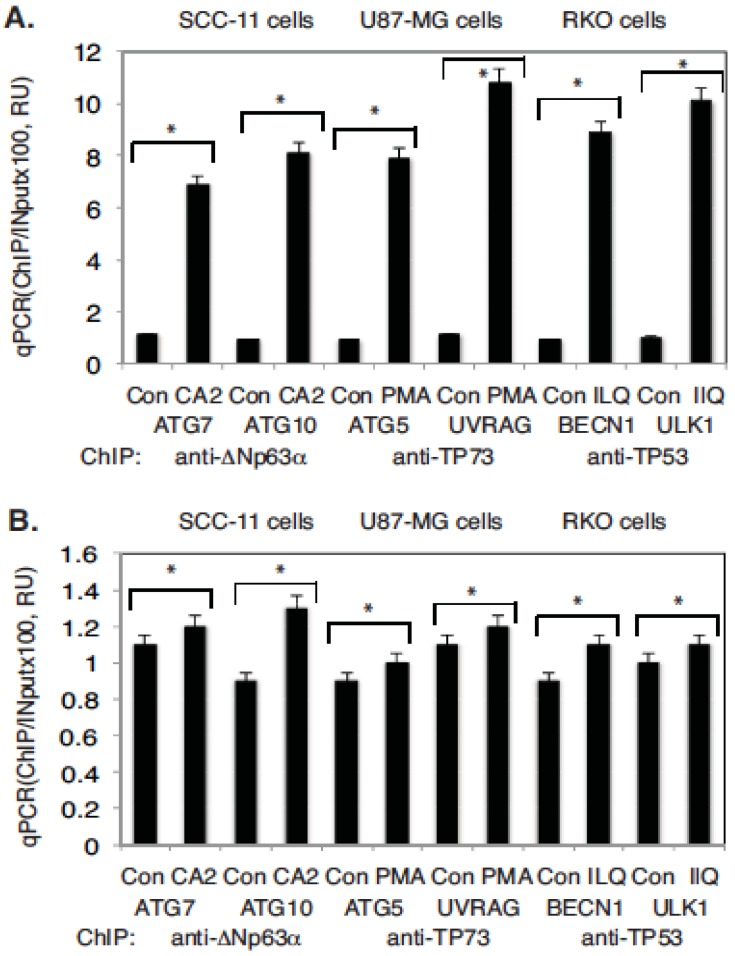
TP53 family members bound to the tested autophagic gene promoters upon exposure to the indicated marine drugs. Tumor cells were exposed to control medium (Con) or medium with chromomycin A2 (CA2, 30 nM), psammaplin A (PMA, 7.5 µM), and ilimaquinone (ILQ, 10 µM) for 12 h. Chromatin imunoprecipitation (ChIP) of the TP53, TP63, and TP73 (using indicated antibodies) bound to the specific regions (**A**) and nonspecific regions (**B**) of the indicated autophagic gene promoter sequences. Quantitative real-time qPCR experiments were performed in triplicate with ±SD as indicated (* *p* < 0.05). The amount of immunoprecipitated-enriched DNA in each sample (ChIP) is represented as a signal relative to the total amount of input chromatin DNA (Input) using the same primers multiplied by 100. Assays were performed using three independent biological experiments in triplicate.

We then investigated whether TP53 family members are able to bind to the selected autophagic promoters in tested tumor cells upon exposure to marine drugs. These abilities were assessed using the chromatin immunoprecipitation (ChIP) analysis, as described elsewhere [30]. Tumor cells (SCC-11, U87-MG, and RKO) were treated with selected marine drugs (CA2, PMA, and ILQ) or control medium for 12 h. ChIP analysis was performed with the ChIP-grade antibodies against ΔNp63α, TP73α, and wild type TP53. We found that the exposure of tumor cells to selected anticancer marine compounds led to an increased enrichment in the preferential binding of TP53 family transcriptional factors to the selected autophagic gene promoters (Figure 5A) via recognition of the TP53/TP73/TP63 responsive elements found in the specific regions of these gene promoters (Supplementary Figures S1–S6). However, non-specific regions of these gene promoters that lack defined responsive elements for TP53 family members, and that are used as negative controls, failed to bind these transcriptional proteins (Figure 5B).

**Figure 6:**
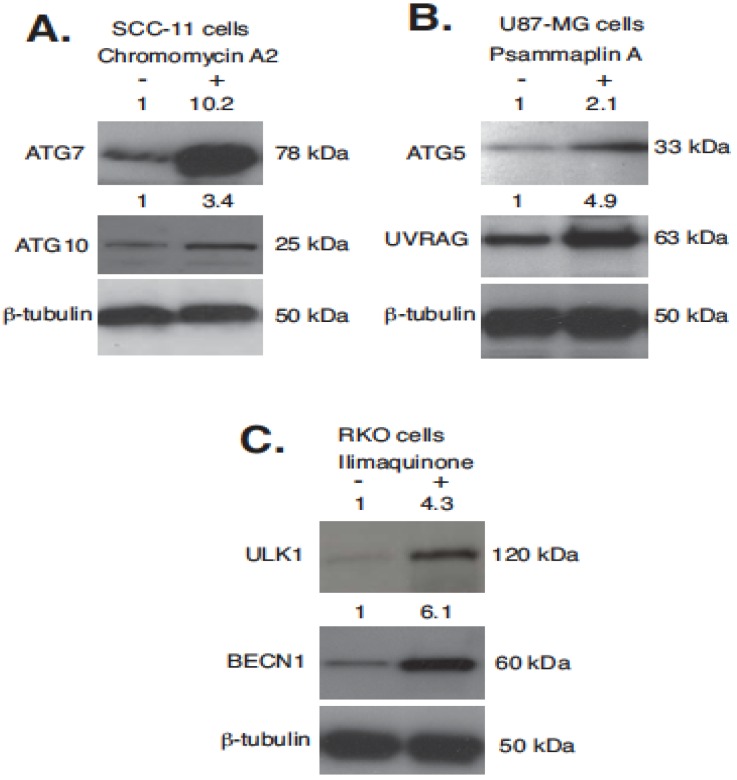
Expression of autophagic proteins (ATG7, ATG10, ATG5, UVRAG, BECN1, and ULK1) in human tumor cells upon exposure to indicated marine drugs. Tested tumor cells were exposed to control medium (−) or medium with chromomycin A2 (+, 30 nM, **panel A**), psammaplin A (+, 7.5 µM, **panel B**), or ilimaquinone (+, 10 µM, **panel C**) for 16 h. Total protein lysates were analyzed by immunoblotting with indicated antibodies against ATG7, ATG10, ATG5, UVRAG, ULK1, and BECN1. Level of β-tubulin served as a loading control.

We then showed that the tested marine drugs (CA2, PMA, and ILQ) were able to induce the expression of autophagic proteins involved in autophagy signaling in human tumor cells (SCC-11, U87-MG, and RKO) in vitro (Figure 6). We found that SCC-11 cells exposed to 30 nM CA2 exhibited a dramatic increase in the ATG7 and ATG10 protein levels, while the treatment of U87-MG cells with 7.5 µM PMA led to a substantial increase in the ATG5 and UVRAG protein expression, and 10 µM ILQ induced the marked expression of ULK1 and BECN1 proteins in RKO cells (Figure 6).

### 2.5. Silencing of TP53 Family Members Modulated the Expression of Autophagic Genes in Tumor Cells

**Figure 7:**
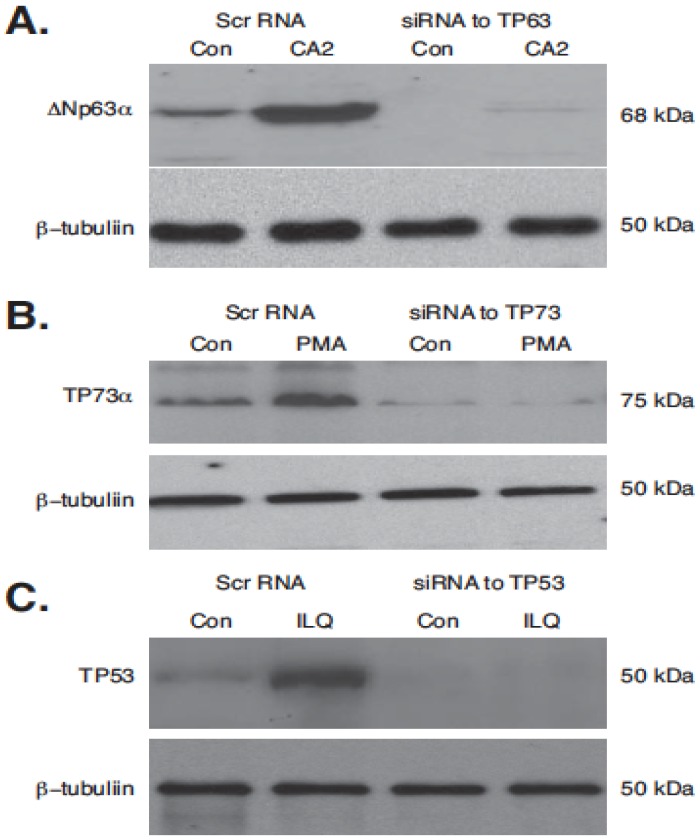
Silencing of TP53 family members by siRNA upon exposure of tumor cells selected marine drugs. Tumor cells (SCC-11 in **panel A**; U87-MG in **panel B**; and RKO I **panel C**) were transfected with the scrambled siRNA (Scr RNA) and siRNA against TP63 (**panel A**), TP73 (**panel B**), and TP53 (**panel C**) for 48 h, and were subsequently treated with control media (Con) and indicated marine drugs (CA2, 30 nM, PMA, 7.5 µM, and ILQ, 10 µM) for an additional 16 h. Expression of TP53 family members was examined by immunoblotting with indicated antibodies. Level of β-tubulin served as a loading control.

**Figure 8:**
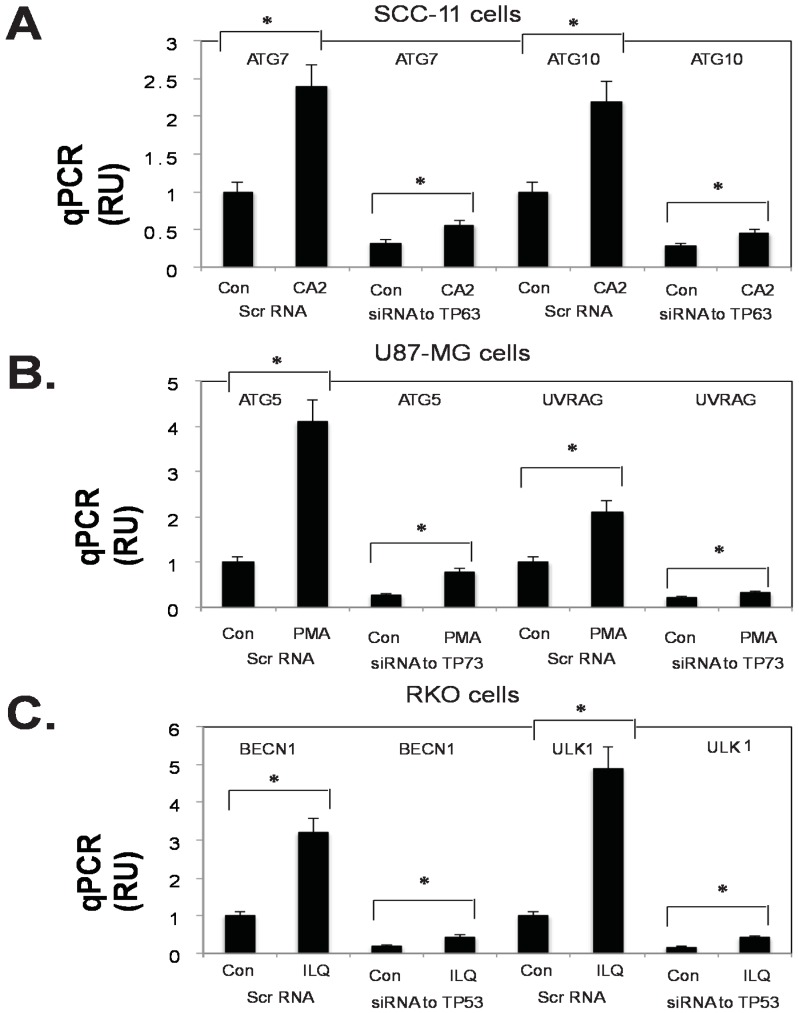
Silencing of TP53 family members by siRNA modulate the transcription of tested autophagic genes (*ATG7*, *ATG10*, *ATG5*, *UVRAG*, *BECN1*, and *ULK1*) in tumor cells treated with selected marine drugs. Tumor cells (SCC-11 in **panel A**; U87-MG in **panel B**; and RKO I **panel C**) were transfected with the scrambled siRNA (Scr RNA) and siRNA against TP63 (**panel A**), TP73 (**panel B**), and TP53 (**panel C**) for 48 h, and were subsequently treated with control media (Con) and the indicated marine drugs (CA2, 30 nM, PMA, 7.5 µM, and ILQ, 10 µM) for an additional 16 h. QPCR analysis of gene targets in SCC-11 cells, U87-MG cells, and RKO cells. Three independent qPCR assays were performed in triplicate (± SD are indicated. * *p* < 0.05).

Finally, we examined whether the silencing of TP53 family members (TP53, TP63, and TP73) would affect the transcriptional regulation of selected autophagic genes (*ATG1/ULK1*, *ATG5*, *ATG6/BECN1*, *ATG7*, *ATG10*, and *UVRAG*) in the tested tumor cells (SCC-11, U87-MG, and RKO) upon treatment with marine anticancer compounds (CA2, PMA, and ILQ). We found that in contrast to the scrambled RNA (Scr RNA), siRNA against TP63, TP73, and TP53 markedly decreased the protein levels of predominant isoforms ΔNp63α, TP73α, and TP53 in the tested tumor cells (SCC-11, U87-MG, and RKO) even upon subsequent exposure of the tumor cells to the selected marine compounds (CA2, PMA, and ILQ), as shown in Figure 7. Using the qPCR assay, we further found that the expression of autophagic genes is induced in SCC-11 cells (ATG7, ATG10), in U87-MG cells (ATG5, UVRAG), and in RKO cells (BECN1, ULK1) upon treatment with CA2, PMA, and ILQ in the presence of the scrambled RNA (Figure 8).

However, the expression silencing of TP53 proteins led to a dramatic modulation of the *ATG7* and *ATG10* mRNA levels in SCC-11 cells, the *ATG5* and *UVRAG* mRNA levels in U87-MG cells, and the *BECN1* and *ULK1* mRNA levels in RKO cells treated with the selected marine anticancer compounds (CA2, PMA, and ILQ, respectively), as seen in Figure 8. This supports the notion of the direct involvement of various TP53 family proteins in the transcriptional regulation of autophagic proteins in tumor cells (SCC-11, U87-MG, and RKO) upon exposure to CA2, PMA, and ILQ. Taken together, our results provide evidence that selected marine anticancer compounds (CA2, PMA, and ILQ) are able to induce autophagic flux through a transcription-dependent activation of key autophagic genes (*ATG1/ULK1*, *ATG5*, *ATG6/BECN1*, *ATG7*, *ATG10*, and *UVRAG*) by TP53 family members.

## 3. Discussion

As indicated in the Introduction section, autophagy plays a critical role in cancer initiation and progression, providing challenges for current anticancer strategies [5]. Increasing evidence points towards the prognostic significance of autophagy biomarkers in solid tumors, leading to novel chemotherapeutic strategies through which to harness autophagy modulation and promote tumor cell death [2]. Interestingly, several natural anticancer agents were found to exert their anticancer effects by triggering autophagy [31,32,33,34,35,36,47]. Emerging data suggest that autophagy represents a novel mechanism that can be exploited for therapeutic benefit [31,32,33,34,35]. Pharmacologically active natural compounds, such as those from marine, terrestrial plants, and animals represent a promising resource for novel anticancer drugs [37,38,39].

CA2, PMA, ILQ, and cardamonin were previously shown to induce autophagy in various human tumor cells, including melanoma cells, breast cancer cells, and colon cancer cells [41,42,43,44]. Moreover, some of these compounds were shown to affect the expression of TP53 protein in melanoma cells and colon cancer cells [42,43,44]. For example, CA2 reduced melanoma cell proliferation via cell cycle arrest in the G0/G1 phase, and expression of cyclins [41]. Additionally, melanoma cells displayed some autophagosome-like structures and exhibited an increased expression of LC3-A and LC3-B proteins upon exposure to CA2 [41]. Treatment of breast cancer cells with PMA showed specific cellular responses, including cell cycle arrest and apoptosis, as well as induction of autophagy protein expression, such as LC3B, and DRAM1, a TP53-induced protein, as indicated in [42]. ILQ and ethylsmenoquinone (EMQ) were reported to activate the TP53 pathway in colon cancer cells [43]. Both marine compounds (ILQ and EMQ) were found to stabilize the TP53 protein through a TP53 phosphorylation at the S15 position in HCT116 and RKO colon cancer cells, leading to upregulation of the CDKNA1 expression, thereby suppressing the proliferation of colon cancer cells [43]. Moreover, autophagic flux was elicited by these compounds, as indicated by LC3 puncta formation and LC3-II turnover [43]. Finally, cardamonin (2′,4′-dihydroxy-6′-methoxychalcone) from *Alpinia katsumadai Hayata* (Zingiberaceae) inhibited cell proliferation, induced G2/M phase cell cycle arrest, and enhanced autophagy in human hepatocellular carcinoma HCT116 cells, and these processes were regulated by TP53 [44].

The purpose of this study was to further examine the role of TP53 family members in the autophagic process induced by the selected marine anticancer compounds (CA2, PMA, and ILQ). We found that all tested marine compounds markedly decreased the viability of tumor cells selected for these studies (SCC-11, U87-MG, and RKO). Since these cell lines were previously reported to predominantly express TP63 (ΔNp63α), TP73 (TP73α), and TP53 [27,46,47], we further found that CA2, PMA, and ILQ induced the expression and phosphorylation of TP53 family members in these tumor cell lines (SCC-11, U87-MG, and RKO). Specific genotoxic stimuli are known to promote a series of reversible post-translational modifications of the TP53 protein including multisite phosphorylation events (e.g., S15 and S46) of the transactivation domain (N-terminus) leading to activation of TP53 as a transcriptional factor [48]. The phosphorylation at the S15 and S46 positions could stimulate association of TP53 with critical chromatin-associated proteins, such as histone/lysine acetyltransferases, as reviewed in [48]. These protein associations resulted in the TP53 protein stabilization, thereby permitting subsequent stimulation of transcription, as indicated in [28,48]. Similarly, phosphorylation of ΔNp63α at the S385 position and TP73 at the Y99 position were also found to be critical for the transcriptional role for ΔNp63α and TP73, respectively [10,28,30,46].

Therefore, our observations that ILQ could induce the phosphorylation of TP53 at the S15 and S46 positions, as well as that CA2 and PMA could induce the phosphorylation of ΔNp63α at the S385 position, and TP73 at the Y99 position, respectively, might reflect their roles in transcriptional activation of downstream target genes, as indicated in [10,28,30,46,48].

We next found that CA2, PMA, and ILQ induced a transcriptional activation of autophagic target genes (e.g., *ATG7*, *ATG10*, *ATG5*, *UVRAG*, *BECN1*, and *ULK1*) and autophagic flux detected by the LC3BI/LC3BII shift. Moreover, the treatment with CA2, PMA, and ILQ led to an induced expression of these autophagic genes, tested by qPCR assay, luciferase promoter assay, and ChIP assay to determine the binding of TP53/TP63/TP73 proteins to the specific promoters of the tested downstream gene targets. Finally, we found that silencing of TP53 family proteins with siRNA modulated the induction of autophagic gene expression in tested tumor cells upon exposure to CA2, PMA, and ILQ. Our observations thus supported the notion that TP53 family proteins, and their activation via phosphorylation, could play a decisive role in transcriptional regulation of autophagic target genes in the tested tumor cells upon exposure to CA2, PMA, and ILQ. Therefore, these findings enhanced our understanding of the molecular mechanisms underlying the autophagic response of certain tumor cells to the selected marine anticancer compounds.

The autophagy gene (*ATG*) encoded proteins are needed for the induction of autophagy, vesicle generation, maturation, and the recycling of autophagosomes [49]. These proteins combine into four functional groups; a protein serine/threonine kinase complex that responds to upstream signals (ATG1/ULK1, ATG13, ATG17) and initiates an induction of autophagy; a lipid kinase signaling complex that mediates vesicle nucleation (ATG6/BECN1, ATG14, AMBRA1, VPS34, VPS15, and UVRAG), and vesicle elongation (ATG3, ATG4, ATG5, ATG7, ATG10, ATG16, and LC3); two ubiquitin-like conjugation pathways that mediate vesicle expansion (ATG8 and ATG12); and a recycling complex that mediates the disassembly of ATG proteins from matured autophagosomes (ATG2, ATG9, ATG18). Our results showed that in tested tumor cell lines of various origins, which predominantly express distinct members of the TP53 family, certain intermediates of autophagic pathway are transcriptionally regulated, thereby contributing to the induction (ATG1/ULK1 in RKO1 cells), vesicle nucleation (ATG6/BECN1, UVRAG1 in U87-MG cells and RKO cells), and vesicle expansion (ATG5, ATG7, and ATG10 in U87-MG cells, and SCC-11 cells).

Interestingly, other marine anticancer compounds were reported to induce an autophagic response in human tumor cells. For example, Helenalin, a sesquiterpene lactone; in addition to inducing cell cycle arrest and apoptosis, it also increased the levels of the autophagic markers [50]. These studies also indicated that caspase activity was essential for autophagic cell death, as well as emphasized a role for the NF-κB p65 transcriptional factor in these processes [50].

Araguspongines C from the marine sponge *Xestospongia* sp. was found to induce autophagic cell death in HER2-overexpressing BT-474 breast cancer cells leading to a vacuole formation and increasing levels for autophagy markers (e.g., LC3A/B, ATG3, ATG7, and ATG16L) in human breast cancer cells [40]. Ovothiol A, from *Paracentrotus lividus* oocytes, induced the formation of autophagic vacuoles and expression of specific autophagic molecular markers, LC3B-I, LC3B-I to LC3B-II conversion, and BECN1 in human liver carcinoma Hep-G2 cells [51]. Viriditoxin from *Paecilomyces variotii* fungus, which is associated with the jellyfish *Nemopilema nomurai*, increased autophagic cell death in human prostate LNCaP cells by induction of several autophagy-related proteins, such as LC3B, ATG5, ATG7, and BECN1, as described in [52]. Tephrosin was found to induce the formation of acidic vesicular autophagic organelles, as well as increase the LC3B-II/LC3B-I ratio [53]. Citreoviridin (CIT), a mycotoxin from fungal species, was found to induce autophagosome formation and increase the expression of LC3B-II in human liver cancer HepG2 cells [54]. Inhibition of autophagosome formation attenuated CIT-induced apoptosis, indicating that CIT-induced apoptosis was autophagy-dependent [54]. Monanchocidin A (MonA) is a novel alkaloid recently isolated from the marine sponge *Monanchora pulchra*, as indicated in [45]. MonA was shown to induce autophagy at lower concentrations, and lysosomal membrane permeabilization at higher concentrations [45]. The natural product peiminine represses colorectal carcinoma tumor growth by inducing autophagic cell death [55]. Peiminine enhances the autophagic flux by repressing the phosphorylation of mTOR [55]. Silencing of *ATG5* expression greatly reduced the peiminine-induced cell death in wild-type HCT-116 cells, while treating BAX/BAK-deficient cells with peiminine resulted in significant cell death, suggesting a pathway independent of classical apoptosis [55]. Thus, accumulating evidence points to the importance of further understanding of molecular mechanisms underlying upstream regulation of autophagic signaling in tumor cells upon various anticancer compounds from marine organisms, in order to broaden the array of potential chemotherapeutic tools against human cancers.

## 4. Materials and Methods

### 4.1. Cell Lines, Reagents, and Antibodies

We used human glioblastoma U87-MG cells (HTB-14, expressing the wild type TP53, mutated CDKN2A, CDKN2C, and PTEN) and human colon carcinoma cell line RKO (CRL-2577, expressing wild type TP53 and urokinase receptor (u-PAR), but lacking nuclear thyroid receptor), which have been recently purchased for these studies from the American Type Culture Collection (Manassas, VA, USA), as well as human head and neck squamous cell carcinoma cells SCC-11 (expressing wild type TP53, wild type TP63 is amplified, and ΔNp63α is overexpressed, obtained from the Head and Neck Cancer Research Division at the Johns Hopkins University School of Medicine, Baltimore, MD, USA). All cell lines were authenticated by a short tandem repeat profiling analysis using the AmpFISTR Identifier PCR Amplification Lit (Applied Biosystems/Life Technologies, Carlsbad, CA, USA) with the following markers: Amelogenin X, CSF1PO, D13S317, D16S539, D5S818, D7S820, THO1, TPOX, and vWA at the JHMI Fragment Analysis Facility. Cells were maintained in Modified Eagle’s Medium (MEM, Mediatech Inc., Manassas, VA, USA), supplemented with 10% fetal bovine serum (FBS; HyClone/GE Healthcare, Logan, UT, USA), l-glutamine (2 mM), and 1% penicillin/streptomycin (Mediatech Inc., Manassas, VA, USA).

We used Psammaplin A (Bisprasin, *N*,*N*′′-(dithiodi-2,1-ethanediyl) bis [3-bromo-4-hydroxy-α-(hydroxyimino)-benzenepropanamide, isolated from the Psammaplinaplysilla sponge, P8749, Sigma-Aldrich, St. Louis, MO, USA), Ilimaquinone (3-[(Decahydro-1β,2β,4aβ-trimethyl-5-methylene-1-naphthyl) methyl]-2-hydroxy-5-methoxybenzoquinone, isolated from the Hawaiian sponge, Hippospongia metachromia, I7146, Sigma-Aldrich), and Chromomycin A2 (aburamycin from Streptomyces aburaviensis, CAS 6992-70-7, Santa Cruz Biotechnology, Santa Cruz, CA, USA). Bafilomycin A1 (an inhibitor of vacuolar H+-ATPase that blocks late-phase autophagy, e.g., LC3B-I-to-LC3BII conversion [29,30]) was obtained from Sigma-Aldrich (B1793).

We used the following antibodies against total TP53 (sc-81168), phosphorylated (p)-S46-TP53 (sc-101764), TP73α (sc-7238), p-Y99-TP73 (sc-101769), and ISG20L1 (M-16, sc-243112) were all obtained from Santa Cruz Biotechnology; against TP73α (ab14430, ChIP-grade), ATG1/ULK1 (ab71059), ATG5 (ab77580), ATG6/BECN1 (ab62472), ATG7 (ab89775), ATG10 (ab64125), and UVRAG (ab70807) were purchased from Abcam (Cambridge, MA, USA). Antibodies against ΔNp63 (anti-p40, PC373, residues 5–17 epitope), ATG16L1 (ST1525), β-tubulin (clone AA2, #05-661), goat anti-rabbit (AP307P), goat anti-mouse (AP181P), and donkey anti-goat (AP180P) horseradish peroxidase-conjugated immunoglobulins (IgG) were purchased from the EMD/Calbiochem/Millipore Corporation, Billerica, MA, USA and the antibody against MAP1LC3B (#3868) was obtained from Cell Signaling, Danvers, MA, USA. A custom rabbit polyclonal antibody against a phosphorylated peptide encompassing the ΔNp63α protein sequence (ATM motif, NKLPSV-pS-QLINPQQ, residues 379–392) was prepared, allowing us to recognize phosphorylated (p)-ΔNp63α, as described in [30].

### 4.2. Real-Time Quantitative (q) PCR Assay

To evaluate the expression of various targets in tested tumor cells upon exposure to marine drugs, we employed a real-time qPCR using the following primers: for *ATG1 (ULK1)*, sense (601) 5′-cgaggacaccatcaggctct-3′ (620), and antisense (821) 5′-cccatgtacatggcccccga-3′ (840); for *ATG5*, sense (331) 5′-gaccttctgcactgtccatct (351), and antisense (579) 5′-gactgaaagacctttcattcag-3′ (600); for *ATG6 (BECN1)*, sense (621) 5′-agaccagctggacactcagc-3′ (640) and antisense (821) 5′-tcgagaaggtccaggctgag-3′ (840); for *ATG7*, sense (1101) 5′-agctgagtcatcagtggatc (1120), and antisense 5′-tggcagcagcggaccggctc-3′ (1380); for *ATG10*, (921) 5′-tctcaggatgaacgaaatgt-3′ (940), and antisense (1180) 5′-gtggctcacacctgtaatcca-3′ (1200), and for *UVRAG*, sense (1751) 5′-tcagcattagcccagcctgt-3′ (1770), and antisense (2021) 5′-ggcgagttccacccagtct-3′ (2039). Cells were treated with control media or selected drugs (CA2, PMA, or ILQ) for 16 h and isolated RNA samples were analyzed by real-time qPCR, as described elsewhere [30].

### 4.3. Transfections and Luciferase Reporter Assays

Cells (at 40%–50% confluence) in 24-well plate were transfected for 36 h with the control (empty) pLightSwitch_Prom vector (#S707592), LightSwitch_Pro reporter plasmids (0.1 µg) for the *ULK1* (S707592), *BECN1* (S719414), *ATG5* (S719105), *ATG7* (S703125), *ATG10* (#S707535), and *UVRAG* (S719410) gene promoters (all obtained from SwitchGear Genomics/Active Motifs, Carlsbad, CA, USA) using Fugene HD reagent (Promega, Madison, WI, USA) for 36 h [30]. Cells were also transfected with the RenSP Renilla luciferase plasmid (0.01 µg). Resulting cells were treated with control media or selected marine drugs (CA2, PMA, and ILQ) for an additional 16 h. The LightSwitch Luciferase Assay Kit (SwitchGear Genomics/Active Motifs, Carlsbad, CA, USA) was used to monitor a RenSP Renilla luciferase reporter activity at 480 nM using a luminometer. Data is presented as relative values (RU) to the data obtained from the control samples (cells transfected with the scrambled siRNA and exposed to control media, which was designated as 1). Transfection efficiency was validated using the gWiz High-Expression GFP vector (P040400, Genlantis, San Diego, CA, USA). A total GFP fluorescence was measured (a linear curve was generated with the increasing concentrations of the GFP plasmid) using a fluorescence plate reader. Luciferase activity of each sample was measured and corrected for total GFP.

### 4.4. Chromatin Immunoprecipitation (ChIP) Assay

5 × 10^6^ cell chromatin equivalents were immunoprecipitated with 5 µg of the antibody against ΔNp63α (PC-373, EMD/Millipore) ChIP-grade antibody against wild-type TP53 (GAH-112, Qiagen, Gaithersburg, MD, USA), and ChIP-grade TP73 antibody (#39941, Active Motif, Carlsbad, CA, USA), as described elsewhere [30]. The ChIP-grade normal rabbit immunoglobulin (IgG, ab37415, Abcam, Cambridge, MA, USA) was used, as a negative control. Enriched DNAs were used for real-time qPCR assays. Specific regions for the *ATG1/ULK1* promoter (Figure S1) sense, (-1850) 5′-GGTGCAATCTTTGCTCTTCT-3′ (-1831), and antisense, (-1520) 5′-CCCTTCCAGCTCGGTGGCTT-3′ (-1501); for the *ATG5* promoter (Figure S2), sense, (-1900) 5′-CAGGGTCTCTCTCTGTTACC-3′ (-1881), and antisense, (-1669) 5′-CCCAAAGTGCTGGGATTACA-3′ (-1651); for the *ARG6 (BECN1)* promoter (Figure S3), sense, (-600) 5′-ATCCGCCCGCCTCGGCCTCC-3′ (-581), and antisense, (-520) 5′-GTTCTGAGATGGAGCCTTGC-3′ (501); for the *ATG7* promoter (Figure S4), sense, (-1250) 5′-TCTGCTATTGCACGGTTCCT-3′ (-1231), and antisense, (-1000) 5′-TTTCACCGTTTTAGCCGGGA-3′ (-981); for the *ATG10* promoter (Figure 5), sense, (-1070) 5′-ATAATCTAAATTGGCAGCTA-3′ (-1051), and antisense, (870) 5′-CAGTCACCCCCTTCCTCCAG-3′ (-851); and for the *UVRAG* promoter (Figure S6), sense, (-1900) 5′-AGTGACTCCTTTCTCAACAA-3′ (-1981), and antisense, (-1670) 5′-TACTATATGCCAGGTCCTGT-3′ (-1651).

Non-specific regions were amplified with the following primers: for the *ATG1/ULK1* promoter (Figure S1) sense, (-1350) 5′-TGCACGTGGTGAAAGCCATT-3′ (-1331), and antisense, (-1220) 5′-TGGCTGCCGGCGGTGTGACT-3′ (-1201); for the *ATG5* promoter (Figure S2), sense, (-1500) 5′-CTACCATTATATTTTACTAT-3′ (-1481), and antisense, (-1370) 5′-GCACCTTAATCCCACAAGCT-3′ (-1351); for the *ATG6 (BECN1)* promoter (Figure S3), sense, (-200) 5′-GGAGCCTCCCCATTCTCTGC-3′ (-181), and antisense, (-120) 5′-CCCAGCCCGGCCTCTGGGGG-3′ (-101); for the *ATG7* promoter (Figure S4), sense, (-500) 5′-GAATAACTTTATCTCACTGA-3′ (-481), and antisense, (-370) 5′-GCCACCCTGATGGCCCCTGT-3′ (-351); for the *ATG10* promoter (Figure 5), sense, (-1550) 5′-CATTCTTTGCCATGAGGGAT-3′ (-1531), and antisense, (-1420) 5′-TTTAAATTAACATGGTGGTT-3′ (-1401); and for the *UVRAG* promoter (Figure S6), sense, (-1650) 5′-GTGGGCACTTTACATATGTT-3′ (-1631), and antisense, (-1470) 5′-TGTTCATCCAGGTGGTGGAA-3′ (-1451). The qPCR consisted of 40 cycles of 94 °C for 30 s, 60 °C for 30 s, and 72 °C for 30 s using Taq DNA polymerase (Invitrogen, Carlsbad, CA, USA). The ChIP qPCR values and Input qPCR were normalized for *GAPDH* qPCR values. Input qPCR values were designated as 1. Data is presented as ChIP-qPCR/Input qPCR ratios [30].

### 4.5. Small Interfering (si) RNA and Transfection

Cells (40%–50% confluence) in 6-well plates were also transfected with 20 nM (final concentration) of the scrambled siRNA (Silencer^®^ Negative Control No. 1 siRNA, AM4636), and with TP53 siRNA (sc-29435), TP63 (sc-38488), or TP73 siRNA (sc-36167) using 10 µL of Lipofectamine SiRNAMAX reagent (Invitrogen/Life Technologies, Carlsbad, CA, USA) for 48 h. Transfection efficiency was validated using a Silencer^®^ Cy™3-labeled Negative Control No. 1 siRNA (AM4621, Life Technologies, Carlsbad, CA, USA), as recommended by the manufacturer. Post-transfection, cells were exposed to the control medium, and with indicated marine drugs (CA2, PMA, ILQ) for an additional 16 h. Each experiment was performed 3 times in triplicate.

### 4.6. Cell Viability Assay

Cells in 96-well plates were incubated in serum-free medium with 5 µg/mL of 3-(4,5-dimethyl thiazol-2-yl)-2,5-diphenyl tetrazolium bromide (MTT assay, ATCC 30-1010K) in the dark at 37 °C for 4 h, as previously described [29,30]. Lysed cells were incubated at 37 °C for 2 h and the measurements (A_570_ nm to A_650_ nm) were obtained on a Spectra Max 250 plate reader (Molecular Devices, Sunnyvale, CA, USA). Each assay was repeated three times in triplicate.

### 4.7. Statistical Analysis

Difference between two groups or more than two groups was analyzed by the Student’s *t*-test, and one-way ANOVA test. The levels of significance were set at *p* ≤ 0.05.

## 5. Conclusions

Overall our data showed that the selected marine life-derived compounds (Chromomycin A2, Psammaplin A, and Ilimaquinone) induced expression of several autophagic signaling intermediates in human squamous cell carcinoma, glioblastoma, and colorectal carcinoma cells in vitro through a transcriptional regulation by TP53 family members. Thus, accumulating evidence illustrates the importance of further understanding of the molecular mechanisms underlying upstream regulation of autophagic signaling in tumor cells upon various anticancer compounds from marine organisms in order to broaden the potential chemotherapeutic tools against human cancers.

## Supplementary Materials

The following are available online at www.mdpi.com/1660-3397/14/8/154/s1, Figures S1–S6: Schematic representation of the selected autophagic gene promoters.
