# Supplementary material for: Tumor Protein (TP)-p53 Members as Regulators of Autophagy in Tumor Cells upon Marine Drug Exposure
Source: Mar Drugs. 2016 Aug 16;14(8):154. doi: 10.3390/md14080154 (PMC4999915; doi:10.3390/md14080154)
Supplement: Supplementary file 1 [file marinedrugs-14-00154-s001.pdf]

# Supplementary Materials: Tumor Protein (TP)-p53 Members as Regulators of Autophagy in Tumor Cells upon Marine Drug Exposure

**Edward A. Ratovitski**

**Figures S1–S6. Schematic representation of the selected autophagic gene promoters.** Promoter sequences were obtained from the UCSC website (http://genome.ucsc.edu/) and analyzed using the TFSEARCH software (http://mbs.cbrc.jp/research/db/TFSEARCH.html). TP63 responsive sequences were defined manually using the previously reported cognate sequence. Consensus sequences for the specific transcriptional factors were highlighted in bold with the grey background and boxed. The sequences used to amplify the ChIP-precipitated DNA are underlined.

−1900 GCACGTAGCC ACCTGTTCAC TCATTTTTCT CTTGCAATTT TGCCACGGGT
−1850 GGTGCAATCT TTGCTCTTCT TGTCCCCCAC CCCTAAGCGC TGGGAGCTCC
−1800 TG**CAGGCGGG TCTTGTTCTC ATG**CTGTCAA CTGCAGCACT TACCTCGCAG
**TP53**
−1750 CGTGTGCTCT CTGGGCAGGC CGGTGTGCAG CAGGCACTCC AGGATGTGAG
−1700 TGAATGCTAT GGCTGACTAC GGGGCACTGT GGAACCTCTT TATTGGGAAA
−1650 ACAAA**CATGG TGGGCCAGG**A GACCTCTTGT CCCTTCCAGC TCGGTGGCTT
**TP53**
−1600 **CTGGCCCCCA GTCCAGG**GTC GGGAGTCTGT GTGTCCTGCG GCCTGGAGGA
**TP63**
−1550 TGAGCTGGGT GCAGGTAAAA GGCATCCAGG A**CTTGCTGAT CCAGCCCAGG**
**TP63**
−1500 GCAT**CAGGCT GTCCAGCTGC TGG**GCTGCTC TGGGAGGCAG CATGGAGGGT
**TP63**
−1450 CCTGGCTGAA GTGTGTGTTT CTGGGCTGTT ACTTTAGGAC TGTACTGAAG
−1400 GCTGGGTTGG TGAGCATTGT TTAGAAT**CAT GCATTTGCAA AAGCAAG**TCT
**TP53**
−1350 TGCACGTGGT GAAAGCCATT CCAACTAGCG CGCAACGTGT ACGGTGAACA
−1300 GCACTAACTT TTCCCCGGTC CTCTCTCCCA GAGCAATCGC TGTTGGCTGT
−1250 GTCTTCTGTG AGTGAGCCCC CGGGCTCTTT TGGCTGCCGG CGGTGTGACT
−1200 TTGGACACAT TCTCCAACTT TTCTCCGCCT TGATTTCCTC ATCTGTAAAA

**Figure S1.** ATG1/ULK1 promoter.

−2000 **CAGGAAGTTG AATTTTAAGT CAAATCAAG**T AATCACCTTT CACCTATTGG

**TP73**

−1950 GTTTCTTCAT ACATAG**TTTC CCCCAAA**ACA ATTTTTTTTT TTTTTTGAGA

**STAT**

−1900 CAGGGTCTCT CTCTGTTACC TAGACTGCAG TGATACAATC ATAGCTCACT

−1850 GCAACCTTGA CCTCCTGGGC T**CATGTAATC CTCCTTCCTC AAG**CACTGGA

**TP53/TP73**

−1800 GTAGCTGGGA CTGTAGGCG**C ATGCCACCAT G**CCCTCCTAA TTTTTGTGTT

**TP53**

−1750 TTTTGTAGAG ATGTGGTCTT GC**CATGTTGT GCAGGCTGGT CTTCAAG**TCC

**TP53/TP73**

−1700 TGGGCT**CAAG CTATCCTCTT ACCTG**GGCCT CCCAAAGTGC TGGGATTACA

**TP63**

−1650 GG**CAAGAGCC ACTGTGCCTG G**TCCCAACAT TTTGTTAGGA AATAATTTTG

**TP63**

−1600 AACACACAGC AAAGCTGAAC AAATTTTACA GTGAATACCC ATTTACAATG

−1550 AACCTAGATT CTACCATCTA GATTCTA**CCA AT**ATATTTTA CCATTATATT

**NF-Y**

−1500 CTACCATTAT ATTTTACTAT ATTTGGTTAA TCACATATTT ATCCATCTAT

−1450 GCGCCCCTAA ATCCATCTTA TTTTTTAGAT ACATTTTAAA GGAAATTTTA

−1400 TATACTTTTG TCTGACACTG GGAAGGCAAT GCACCTTAAT CCCACAAGCT

−1350 TTCTTACAGA GGCTAATAGC TGTTTACTGG GGACACTGTA GTGAGGATTT

−1300 CTGCATTGAG TAGGTGAAGA GGACCATAAC TGTGGCTTTC AAACTTTTCT

−1250 GACTGCTACC CTTACTAAGA AATGTATTTC ACATTCCTAT CCAGTGTACA

**Figure S2.** ATG5 promoter.

−700 GATTACAGGC GCACGCCA**CC AAT**GCCAGCT AATTGTATTT TTTAGTAGAG

**NF-Y**

−650 ACGGGATTTA ACCATTTTGG C**CAGGCTGGT CTCGAACTCC TGACCTTG**TG

**TP63**

−600 ATCCGCCCGC CTCGGCCTCC CAAAGTGCTG GAATTA**CAAG CGTGAGCCAC**

**TP53**

−550 **CATG**CCCGGC CTTTTGTTGT TGCTGTTGTT GTTCTGAGAT GGAGCCTTGC

−500 CCTGTCGCCC AGGCTGGAGT GCAGTGGCCC GATCTCGGCT CACTGCAACC

−450 TCCACCTCCC AGGTTCAAGC GATTCTCCTG CCTCAGCCTC CCGAGTAGCT

−400 GGGATTAAGC TGGGATTATA GGCGTGCCCC ACCACGCCCG GCTAGTTTTT

−350 GTATTTTTAG TAGAGACGGG GTTTCACTGT GTTGGC**CAGG CTGGTCTCGA**

−300 **ACTCCTG**A**CC TCACGTGAT**C CGCCCTCCTC GGCCTCCC**CA AGTGCTGAGA**

**TP63** **SREBP-1** **TP63/TP73**

−250 **TTACAGG**CGT GAGCCACCGC GCCCGCCGCC CCCTGAATTT AGAGAATAGC

−200 GGAGCCTCCC CATTCTCTGC GGCCTTGGCT CCTACACTTC CCGTGGTAAC

−150 CTTGTTCATC CGCTGAAGCC CGCTGCTTTT CCCAGCCCGG CCTCTGGGGG

−100 CCGCTGCCGG GCCTGTGAGC CTGTGGAC**CA GGAGCTCCTG CTGCCTG**CGT

**TP63**

−50 AGCGTCACGT CCGGTCTCGG CGGAAGTTTT CCGGCGGCTA CCGGGAAGTC

**Figure S3.** ATG6/BECN1 promoter.

-1300 CATCTGCGGA TGTTTTGGTC AGAGTGATCT CACTATTGCA ATAGTCTCCC
-1250 TCTGCTATTG CACGGTTCCT TCTCTCCCAC CTCTCTCACT TCTTGCAGTA
-1200 ATCCTTTCTT TTTTTTTTTT TTTGAGACGG AGTCTCGCTC TGTCGCC**CAG**
-1150 **GCTGGAGTGC AGG**TGTGGGA TCTCGGCTCA CTG**CAAGCTC CGCCTCCCGG**
**TP63/TP73** **TP63/TP73**
-1100 **GTTCAGGCCA AT**CTCCTGCC TCAGCCTCCC AAGTAG**CTGG GACTACAGG**C
**NF-Y** **TP63**
-1050 GCCCGCCACC AAGCCCGGCT AATTTTTTTG TATTTTTAGT AGAGACGGGG
-1000 TTTCACCGTT TTAGCCGGGA TGGTCTCGAT CTCCTGACCT CGTGATCCGC
-950 CCGCCTCGGC CTCCCAAAGT GCTGTGATTA **CAGGCGTGAG CCACCGCGCC**
**TP63**
-900 **GGGCCTG**CAG TAATCCTTTC TAATAAGTAT CCTTATTAGT CTGGATTTGT
-850 TTTCTTATTT GCCAGAATCA AACAAACTTC AGATTACGTT CCTGGCCCAA
-800 AAGCCCTTTA ACTTTCAGCA AGCCACCTAA CTTTTCTTAG ATTCTGTTCT
-750 TTCATATATA A**CTTGGGGAT ATGTTTCAGG** ACTCTTGTGA GGATTAGAGA
**TP63**
-700 TGTAAAACCC TGAAAA**CATG GTGGCAAG**TT AATTAATA**CA GGTCATCGCT**
**TP53** **TP63**
-650 **CTTG**TTGTTA TGAGTCTAGA GTGGTATATC AGAGTTAATT TGTGAAAGGC
-600 CTTA**CAGGCC AGACAGAGAA ACTTG**TCATA ATCACTGCTG TATCTTCAAC
**TP63**
-550 ACCTGTCCAG TGCTTTGCAC GCG**CAGGATA TCTAACCAAG** TATTGAGAGG
**TP63**
-500 GAATAACTTT ATCTCACTGA CAGTGAGGAG CTGTGAGAAT GATGGCTGGT
-450 TTAGAAAAGG CAGGTTGGTC ACTGTCGACG TTCACTGGCC TTTTCCTACT
-400 AAAATTCTCA TCTCCTGGCT CTCCACACCT GCCACCCTGA TGGCCCCTGT
-350 GCTGCGTTTG ATGCCGCCTC TCCTGGAGAA TGACCATGGT GATCATTCCT

**Figure S4.** ATG7 promoter.

-1550 CATTCTTTGC CATGAGGGAT ATGATGAGAG GAAGTACATG GAGGTTAACT
-1500 GAGTTATTTC TTTTAGTTTC TTTGTTTTTG GCATGTGAAT TATGAAACTT
-1450 TAAAACACCT CCAGTGCAAA AAAAATTTTT TTTAAATTAA CATGGTGGTT
-1400 GGTGCACTGT GTTTCTAAAA TATGGAGTAC AATGTAAAGC AATGCATTCT
-1350 TTTTTTAAAA ATTTATTGTT ATTTTGAAAA ATTTTTGTAG AGATGAGGTC
-1300 TCACTGC**CTT GCCCATAGCT GG**TCTTGAAC TCCTGGGCTA AAGCGATCCT
**TP63**
-1250 CCTACCTCGG GCTCCCAAAG TGCTGGGATT ACAGGCGTGA GCCACTGCGC
-1200 CCAGCCGCAT TCTTGTTTTC ATCTGTGAAA TACGTTTTTA AGGTAAATAC
-1150 TTAAAGTTGA TGGAGACACC TGGTCTACTT AAGCTCTCAT TCAGAACACA
-1100 CTGCCTTGTG CTTTTAAAAA AATTAAATGG ATAATCTAAA TTGGCAGCTA
-1050 **CCAAT**TAATT TATATCCATA GTTGCTAAAA AATGTTAGTG TAAGTTCATT
**NF-Y**
-1000 TTTATTAGTC GCATCATTTT GAGTTTTAAA CTATTTCACA TGCAGTAAAA
-950 TTTATACCTC TTATGGGTTT TGTCAAACAG TGGTTTAGTC ACCAACACAA
-900 TCA**CGGTATA GAAGCTCTCA AG**CTCCTTTT CAGTCACCCC CTTCCTCCAG
**TP63**
-850 CCCTTTGCCC TGGTCGCCAC TGATCAGTAT TCTCTCCTTT TAAAAACACT

**Figure S5.** ATG10 promoter.

-2000 AGAAAACTAA TACAGCTTAG AAACAGAAAT GTGTTTTCTG TGAAATTCCC
-1950 CCAGATCTTG GCACCACATG AGTTCAATCA GATAATATTT GGGATTTCCC
-1900 AGTGACTCCT TTCTCAACAA ACACCTGATC AAGCGACTCC TTTCTCAACA
-1850 AACACCTGAC CACACGCCC**C AGGCTCCAGC CATG**AGGAAC CACTCAGTGT
**TP53/TP73**
-1800 GTCAGAACAG GAGACAGTGT GACATCTTTG TGATCCAGCT CTGAGAAAAG
-1750 ATC**CAAGGTG TTTTTCAGG**C TTCTGAACAC CCTGGAAAGA TAGTATGCTT
**TP73**
-1700 ACATTTTGTT TAAAAAATTT AAAATGCAAC TACTATATGC CAGGTCCTGT
-1650 GTGGGCACTT TACATATGTT GATTTACTTT AATCCCTACA GCAATCCTAT
-1600 TACTATTTTA AAGCTGAGAG AGGGGAAGTG ACTTGCCTTG GGATACATAG
-1550 CTCAAAGGTG GTAGAGCCAA GATTCCAAAG TGCCTGATTC CTATTGTGTC
-1500 TGACTCTAAA ACACTATGTT CTTTCTGCTA TGTTCATCCA GGTGGTGGAA
-1450 AAATTCAGTC TAGGAGTAAA AAAAGGAAGA TCAACATCCT GCAGAATGAC

**Figure S6.** UVRAG promoter.
